# Circadian Clocks in Fish—What Have We Learned so far?

**DOI:** 10.3390/biology8010017

**Published:** 2019-03-19

**Authors:** Inga A. Frøland Steindal, David Whitmore

**Affiliations:** Department of Cell and Developmental Biology, University College London, 21 University Street, London WC1E 6DE, UK; inga.steindal.12@ucl.ac.uk

**Keywords:** zebrafish, circadian clock, development, DNA repair, non-visual light detection, cell cycle

## Abstract

Zebrafish represent the one alternative vertebrate, genetic model system to mice that can be easily manipulated in a laboratory setting. With the teleost Medaka (*Oryzias latipes*), which now has a significant following, and over 30,000 other fish species worldwide, there is great potential to study the biology of environmental adaptation using teleosts. Zebrafish are primarily used for research on developmental biology, for obvious reasons. However, fish in general have also contributed to our understanding of circadian clock biology in the broadest sense. In this review, we will discuss selected areas where this contribution seems most unique. This will include a discussion of the issue of central versus peripheral clocks, in which zebrafish played an early role; the global nature of light sensitivity; and the critical role played by light in regulating cell biology. In addition, we also discuss the importance of the clock in controlling the timing of fundamental aspects of cell biology, such as the temporal control of the cell cycle. Many of these findings are applicable to the majority of vertebrate species. However, some reflect the unique manner in which “fish” can solve biological problems, in an evolutionary context. Genome duplication events simply mean that many fish species have more gene copies to “throw at a problem”, and evolution seems to have taken advantage of this “gene abundance”. How this relates to their poor cousins, the mammals, remains to be seen.

## 1. Introduction

As we have all noticed by now, the sun comes up in the morning and sets in the evening with some predictability. For those living in London, New York or Tokyo where the light is more or less constant—you are more likely to suffer from some horrible clock-disrupted illness. There is no doubt that possessing a functional clock is key to optimal health, and entrainment of that clock is an essential process. Light is the typical, but far from only, environmental signal that sets the clock, and work on fish has revealed some unexpected aspects of non-visual photoreception, as we will discuss below. In this respect, various fish models offer unique tools with which to study the wide significance of light responsiveness, and this is no more true than in the use of naturally occurring cave populations of fish, in particular the blind Mexican cavefish (*Astyanax mexicanus*) and the Somalian cavefish (*Phreatichthys andruzzii*). These animals, which have evolved in complete darkness, can effectively be viewed as circadian/light responsive “mutants”, and as such offer considerable potential for exploring the global importance of light [1,2,3,4].

Still one of the most useful ways to view the circadian clock system is to employ the “Eskinogram” model, first proposed by Arnold Eskin, in which the clock is broken up into light detection, input pathway, core oscillator and then downstream, clock-regulated output events. In this context, it is probably fair to say that the fish models have not yet contributed significantly to our understanding of the core clock mechanism. However, it is in the areas of input and output events where most significant contributions have occurred from many research groups, along with emerging studies on the ecological adaptations that the clock undergoes in unusual environments [5]. Some of these findings, though far from all, will be discussed in the following sections of this review.

## 2. Peripheral Clocks and the Emergence of Cell Lines

### 2.1. Clocks Everywhere

**Figure 1:**
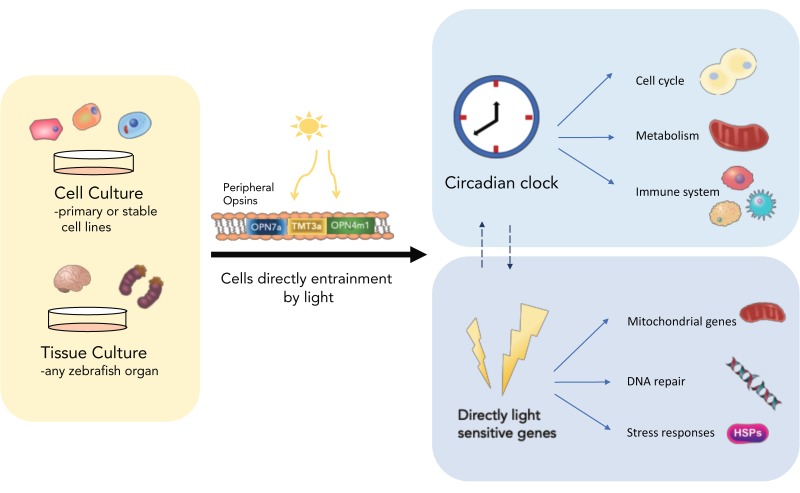
Zebrafish tissues are rhythmic and directly light-responsive. All zebrafish cell types and tissue/organs examined to date are directly light-responsive and do not require a centralised photosensitive structure to turn on light-induced transcription. Cells and organs can be entrained directly by light stimuli through the use of visual and non-visual peripheral opsins. The light signal starts transcription of light-sensitive genes, such as stress responses and DNA repair, as well as the clock genes *per2* and *cry1a*, which sets the circadian clock. The peripherally entrained clock in turn regulates a plethora of downstream cellular processes [14].

The late 1990s saw a relatively fast transition from the classical clock view that circadian pacemakers are restricted to specialized “clock-containing” structures, to the idea of clocks residing in most, if not all cells and tissues [6,7,8,9]. These discoveries, of course, followed on from the initial isolation and cloning of clock genes, which then allowed for their expression patterns to be determined. In mouse, these genes are expressed in most tissues [10,11,12]. Then, with the advent of mammalian cell line experiments, it was shown that mammalian cell lines could produce oscillations in clock gene expression if the cells are synchronized in an appropriate pharmacological manner [13]. Work in zebrafish was occurring in parallel to these mammalian studies, and it was quickly shown, after the cloning of the zebrafish *clock* gene, that oscillations in this clock component occurred in all tissues examined, both in vivo and in vitro [7,8]. There are clearly independent circadian pacemakers within all of the fish tissues that have been examined. These observations were then expanded to include zebrafish cell lines, which show high-amplitude, robust clock rhythms at the transcriptional level [8]. Figure 1 is a simple diagrammatic summary of how we view zebrafish clock organization. 

### 2.2. Global Light Sensitivity

At a very overt level, the big difference between mammalian cell/tissue clocks and those found in fish cells and tissues is that fish cells are directly light responsive [8]. The clock appears to be set by light directly, without any apparent need for eyes or pineal gland, the classical light responsive structures. Now it is clear that most of the studies that have addressed this issue have been performed with tissues or cells in culture, where light sensitivity is fully retained. But it is also apparent that peripheral light responsiveness is retained in zebrafish larval mutants, where either eyes or pineal are missing/defective. This does not mean that the eyes/pineal might not contribute to peripheral tissue light sensitivity, via either neural or hormonal signals, as modulators of this response, but there is no evidence at this time that this either occurs or is necessary. 

This highly decentralized model of fish circadian biology, with independent, light-responsive circadian pacemakers in all tissues and most cells, does appear to hold true for most fish species that have been examined to date [15,16,17]. Although there is a lack of data on some of the really large marine species, which would represent an entertaining project if nothing else. In mammals, of course, the environmental light signal is transmitted to the suprachiasmatic nucleus (SCN) to set a “central” clock, which then plays a role in coordinating the timing of peripheral pacemakers [18]. This level of organization does not appear to be necessary in fish, but again this does not mean that there is not potential interaction between tissue clocks within the fish body. Obviously, a whole variety of hormonal signals, including rhythmic melatonin cues, could also be influencing tissue-specific, daily oscillations. It is interesting that the environmental light signal appears to set the clock to the same phase in all cells and tissues in vitro. Of course, what might occur in vivo is that light sets all of the body clocks to the same phase, and then various hormonal/neural cues apply subtle (or not so subtle) adjustments to this timing, generating tissue-specific phasing of rhythms. In the end, answering this question will require quite sophisticated imaging of organ rhythms in vivo, combined with a clever use of fish mutants, in order to determine the precise phase of various tissue clocks in response to numerous potential entraining signals. Such experiments may well be feasible in zebrafish, due to the small size and relative transparency of the larval body. It is not clear if fish do possess a “central” clock, but equally this idea might be rather redundant and something of a “red herring”. It is clear that there are separate clocks in the brain and pineal gland, as there are in the heart and liver. They can all be synchronized independently, but may interact. There is no doubt that the pineal pacemaker plays a key role in influencing sleep processes and rhythmic behaviour, just as the heart clock plays a role in rhythmic heart physiology [19,20,21]. The ideas of “central” or “master” clock are no longer necessary or useful concepts. The importance of whole-body light sensitivity, however, is clearly important for fish physiology, and will be explored in more detail in Section 3. 

### 2.3. Development of the Clock and Its Relevance

It is clear that the major advantage of using zebrafish as a model system is to study the ontogeny or development of clock function in the earliest stages of embryogenesis. Such experiments are difficult to perform in mammals for obvious reasons, mostly relating to the internal development of the foetus within the mother. The humble zebrafish is robust, cheap, well sequenced and shares similar genetic and organ structures to humans. Furthermore, the zebrafish is a well-established model for studying vertebrate development with transparent eggs being laid in their hundreds every other morning. Zebrafish embryo development is also rapid, with the first 24 h being equivalent to about 1 month of human development. Consequently, zebrafish also have a short generation time. What also makes the zebrafish useful for circadian studies is that they mate just after daybreak, meaning that developmental stage and circadian time are well aligned. Despite these advantages, this whole story marrying development and circadian rhythms got off to a dismal start with the publication of the idea that embryos inherit a sense of circadian phase from their mothers. This is not correct. Subsequent data in fact supports the idea that a circadian pacemaker is indeed present in the early stages of embryo development, with a peak in *per1* gene expression clearly present 27 h post fertilization (hpf) when embryos are raised on a light-dark cycle. However, when raised in the dark, no such molecular clock rhythms are seen at the population level (in contrast to the initial reports). Considerable evidence backs up this theory, with multiple reverse-transcription quantitative polymerase chain reaction (RT-qPCR) experiments in zebrafish, as well as other teleosts, showing that the embryos require light as an entraining signal during the first day of development, to synchronize the temporally dispersed cellular oscillators [22,23]. Embryos are certainly strongly light responsive by 9 hpf, when they are only just beginning the process of gastrulation, and long before any classical light-responsive structures, such as eyes and pineal, have developed. In addition to light, temperature cycles can also entrain this embryonic clock, and similar light-dependent entrainment of a clock in the embryonic pineal gland is essential for early rhythms in *N*-acetyltransferase (NAT)expression and melatonin release [24,25]. 

**Figure 2:**
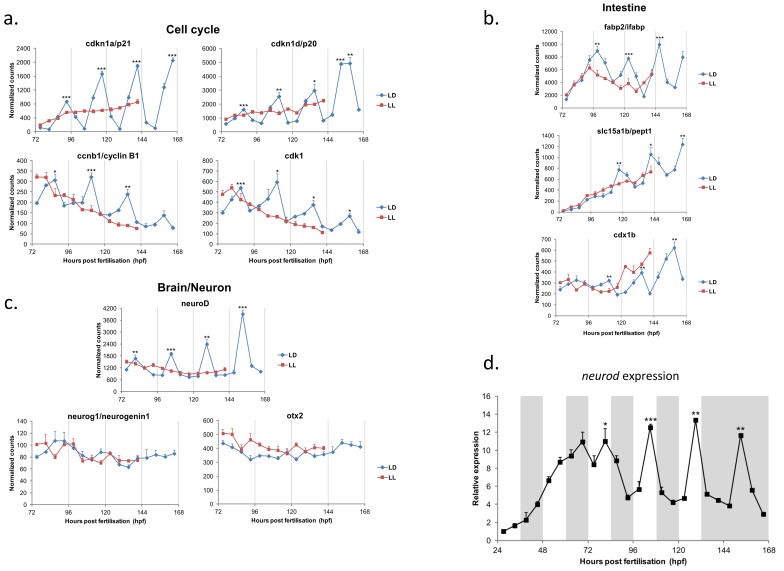
A selection of rhythmic clock-target genes regulated in the early stages of zebrafish larval development. A Nanostring-based gene expression analysis of a wide selection of genes examined between 72–168 h post-fertilization. The selected data shown in panels a, b and c reveals a wide range of genes that show robust oscillations during embryo development, when larvae are raised on a light-dark cycle. Constant light (in red) stops the circadian pacemaker in the embryo, as well as the rhythmic expression in downstream, clock-regulated genes. (**a**) shows that numerous cell cycle regulators have robust transcriptional daily rhythms. (**b**) shows changes in three genes involved in neuro-development and differentiation, with *neuroD* showing very high amplitude rhtyhms. (**c**) shows rhythms in three genes involved in cell fate decisions in the intestine. (**d**) shows how *neuroD* only begins to show robust oscillations from day 4–5 of development onwards. (Taken from Laranjeiro and Whitmore, 2014) [26].

The currently accepted view is that the core molecular clock starts to oscillate very early on in development, probably in all cells of the embryo, but that light exposure is necessary to synchronize each of these randomly phased cellular pacemakers. This is something of an important note to all of those developmental biology labs who still raise their larvae in dark incubators! But what is the clock actually regulating in embryo development and is this story as simple as it seems? To explore this question, larval samples were collected every 6 h from 72–168 h post-fertilization, and then the temporal expression pattern of nearly 100 specifically selected, developmentally critical genes was measured [26]. From this sample, a significant number of regulatory genes showed robust, high-amplitude circadian changes in expression levels. Figure 2 shows some of this embryonic gene expression data under light-dark conditions, and when the circadian pacemaker is stopped using constant light exposure. These downstream targets include numerous cell cycle regulators, discussed below, but also central, cell fate-determining transcriptional regulators, such as *neurod* and *cdx1b*. Both of these proteins play key roles in cell differentiation during development, with *neurod* regulating the early differentiation of neurons and pancreas, and *cdx1b* regulating early endoderm and digestive tract formation, as well as cell fate in the intestine [27]. These results suggest that the clock could play a major role in determining the timing of cell differentiation during development. However, this point remains to be proven and the situation may be more complex, especially in the context of *neurod*. Several aspects of its temporal and spatial regulation raise questions about its specific, clock-regulated role. Firstly, most of these developmental, output genes only begin to show robust daily rhythms from 72–96 hpf onwards. In zebrafish, this is quite late in development and after their recognized major developmental role, which would normally occur within the first 48–72 h. These results indicate that these genes come under clock regulation after their regulatory role in early development is complete. One possibility is that these developmental genes are now being used for a different purpose in later stage larvae and adults than in the early stages of development. In the case of *neurod*, the strongest daily rhythms occur in the retina. Retinal photoreceptor genesis requires precise regulation of cell cycle exit and differentiation, which is aided by *neurod* through the Notch signalling pathway. If one were to look, we might find that adult photoreceptor genesis is clock-regulated in zebrafish. Such results could have interesting implications for the regulation of stem cell niches within adult tissues of many species. 

## 3. The Importance of Light

### 3.1. The Photopigments

Vertebrate photoreception is often thought of as a process exclusively involving the visual system. Although visual light detection using rods and/or cones is obviously important in most vertebrates, non-visual photoreception and the use of non-visual opsins is also important in many critical biological processes, such as seasonality/photoperiodism, circadian entrainment and DNA repair [28,29,30,31,32]. Historically, there was an assumption that there would be one key opsin for non-visual photoreception, underpinning, for example, clock entrainment. Thus, when melanopsin was discovered, many researchers believed that no more non-visual opsins would be discovered (at least not in mammals). However, this view was not to last for long, and as of 2018, due to a considerable improvement in the quality of genome sequencing, we now know there is an immense diversity of non-visual opsins [33]. The non-visual opsins are all seven-transmembrane-domain proteins, like the visual opsins and function using similar mechanisms to those of the classical extra ocular photoreceptors. As opsins belong to the G protein-coupled receptor (GPCR) superfamily of proteins, it follows that opsins may signal and thereby activate light-induced and clock genes, using the classic, well-established downstream pathways. In zebrafish, several reports have implicated the MAPK pathway with light-dependent, transient induction of phosphorylated ERK and MEK [34,35,36,37,38]. Furthermore, pharmacological assays have also pointed to signalling through the phosphoinositide pathway, which interacts with nitric oxide (NO) and the MAPK pathway [39]. Though there are concerns about the reproducibility of these findings, and their validity in general, the nature of this signalling pathway is likely to be very complex, and may include numerous downstream signalling events. 

Phylogenetic studies show that teleost genomes encode 20 different classes of opsins, while reptiles, birds and amphibians also show a high genomic diversity of opsin classes, with 19, 17 and 18 classes respectively [33]. In zebrafish, we find 42 different opsin genes (10 visual and 32 non-visual), currently the highest reported number of opsins in any animal. The expression pattern of these opsins is quite interesting, with usually multiple non-visual opsins expressed in every organ [33]. The internal organs, such as the heart and liver, possess the least, but the brain and retina express almost the “full set”. Non-visual opsins and non-visual light detection is, of course, not exclusive to the lower vertebrates. All three classes of mammals, including eutherians, encode several classes of non-visual opsins [33]. 

The exact function of all of these non-visual opsins remains largely unexplored. In fish, there has been little work to date on the biological relevance of this great diversity of light-detecting molecules, but absorption spectra and tissue-specific expression data have been published [33]. The majority of these opsins have been shown to form functional opsins when expressed in in vitro situations, such as neuro 2A cell lines. Whether this is their primary role in vivo is of course another matter. 

In many respects, it is hard to understand the requirement for such a large number of photopigments, as one would imagine that the role of just detecting light could be performed adequately by far fewer. However, this diversity would certainly ensure a wide range of spectral sensitivity and that “no photon goes undetected”. Presumably there must be some biological value to this. In reality, most of our functional knowledge comes from mouse studies, yet there is still relatively little examination of the role of these opsins in tissues other than the retina. There are some emerging results from rather unexpected tissues, including human adipocytes, suggesting a wide range of light-regulated biology may even exist in humans [40]. Melanopsin (OPN4m), is the most explored non-visual opsin to date and has been implicated in circadian clock entrainment and pupillary constriction in mammals [41,42]. Whether it performs the same circadian role in fish is currently under investigation. Neuropsin (OPN5) has also gained some recent interest, and in 2015, Van Gelder and colleagues showed that neuropsin is critical for photoentrainment in mouse retina [43]. The ultraviolet (UV) light sensitivity of OPN5, and its curious expression pattern in all species where it has been examined, suggest that it may play an interesting role in certain fundamental aspects of cell biology [44].

### 3.2. Light Input Pathway

In zebrafish, the precise nature of the signalling pathways is not yet clear. What is clear however, and has been well-defined, is that light dramatically increases expression of the clock genes *period2* (*per2*) and *cryptochrome 1a* (*cry1a*) in all tissues and cell lines that have been examined [24,28]. PER2 proteins contain a C-terminal CRY binding domain enabling dimerization of PER2 and CRY1a. In turn, the CRY1a protein interacts directly with core clock components, CLOCK and BMAL, blocking their ability to dimerize and thereby repressing transcription by CLOCK:BMAL, providing a likely mechanism for clock resetting [28]. Furthermore, light-induced CRY1a acts to “lock up” or “jam” the clock mechanism, and prevent the molecular core clock from oscillating. It does so in a phase-dependent manner, as CRY1a can only interact with CLOCK and BMAL proteins of course when they are present, which is typically after Zeitgeber Time (ZT) 12. In this way, constant light acts to “stop” the clock at ZT12, and the clock oscillation will not continue again until the light stimulus is removed, and CRY1a protein most likely degrades. This represents a fascinating potential interaction between an hour-glass time measuring system and a circadian pacemaker.

How does light regulate the transcription of these two key genes? Promoter analysis of *per2* and *cry1a* has identified a ‘Light Responsive Module’ consisting of E- and D-box elements spaced close together and in proximity to the transcriptional start site [28,45,46]. This module is also strongly conserved in other *per2* vertebrate genes, including species lacking directly light-sensitive clocks [41,42]. The D-box confers light-driven expression through binding of the thyrotroph embryonic factor (TEF) zebrafish homologue, whilst the E-box directs circadian clock regulation by mediating CLOCK/BMAL activity [11,13]. In addition to TEF, the zebrafish possess an additional 11 D-box binding factors, with nine of them enhanced in the pineal gland, further supporting the involvement of this pathway in the circadian clock mechanism [15].

Although both *per2* and *cry1a* genes possess the light responsive module, their regulation is markedly different. Upon blocking protein synthesis with cycloheximide, the light response using the D-box enhancer is attenuated in *cry1a*, making light-induction of *cry1a* dependent on *de novo* protein synthesis. This is not the case for light-dependent expression of *per2*, which seems to utilise the E-box when protein synthesis is blocked [47,48]. Furthermore, AP-1 enhancer elements have also been implicated in *cry1a* light-driven expression [47]. These results taken together suggests that both these core clock genes use D-boxes to drive their expression, but that multiple other enhancer and control elements ensure that light-driven expression is controlled in a gene promoter-specific manner.

Light sensitivity is not constant over a 24-h period, due to clock-feedback on to the input pathway. Light-pulsing experiments show that light sensitivity in zebrafish is time-of-day dependent, with more than twice the induction of *cry1a* at CT20 compared to CT8 [28]. In turn, the light intensity also impacts the size of the phase shift. Results from phase and intensity response curves demonstrate a strong correlation between light induction of the *cry1a* gene and clock resetting.

Zebrafish has one *per2* gene, in contrast to many other teleost species, such as *Astyanax mexicanus*, where there has been a clear duplication of the *per2* gene. The distinct roles of *per2a* and *per2b* are unclear, but in *A. mexicanus*, the expression patterns in light-dark (LD) and constant dark (DD) differ, indicating that they may have distinct roles [1]. The situation for the multiple *cryptochromes* in zebrafish is equally unclear, where there are at least six distinct *cry* genes. However, it is clear that some of these Cry proteins can act as transcriptional repressors, as in the mammalian clock system [48]. It has also been suggested that Cry4 might act as a photopigment, akin to the situation in Drosophila, but at the minute there is no compelling data to support this hypothesis.

### 3.3. Light Sensitivity in Fish—Light Detection Is Everywhere

Zebrafish and other teleosts, such as the Mexican blind cavefish (*Astyanax mexicanus*) and Medaka (*Oryzias latipes*), have directly light-entrainable tissues [2,49]. For zebrafish, both organs and cells in culture light can entrain the cell endogenous clock, but does this broad light sensitivity impact on other important aspects of cell and tissue biology?

Light is essential for successful development in many teleost species, and the lack of light during development is associated with higher mortality rates and more developmental deformities [32]. In some fish species, like the flatfish *Solea senegalesis*, there is a remarkable 100% mortality in embryos by Day 4 of development when animals are raised in constant darkness [50]. It is quite a common phenomenon in teleosts that light exposure is essential for early survival. Light-regimes and the clock also have an effect on the hatching of larvae. Entrained zebrafish and *S. senegalesis* embryos restrict hatching to a particular time window in the day. Constant light conditions disrupt this timed regulation, with resulting ultradian bouts for zebrafish and 24-h delays or advances in DD and LL respectively for flatfish hatching [1]. Such results have significant implications for commercial fisheries, which often employ constant light conditions to influence early larval growth rates.

Everybody that works with zebrafish is aware that spawning is tightly timed, and that the fish lay eggs just after dawn. This may sound somewhat illogical, as the embryo will undergo DNA replication and rapid cell division at the peak of diurnal UV light exposure, consequently increasing the chance of DNA damage dramatically at this sensitive early stage of development. Perhaps, therefore, it is not surprising that early exposure to light is actually beneficial and, in fact, essential for survival in early embryos. Embryos on the first day of development, raised in DD, show only a 20% survival rate when exposed to a 5 s UV-pulse compared to 85% survival for sibling embryos raised on LD cycles [32]. This increase in survival rate in embryos raised on LD is due to the light-induced expression of DNA-repair enzymes, such as, but not exclusively, *6-4 photolyase*, which is expressed and transcriptionally light-regulated from 6 hpf. During the first 6 h of development, however, zebrafish rely on maternally deposited *6-4 photolyase* transcript. The large quantities of maternally deposited *6-4 photolyase* is not only found in zebrafish, but also in cavefish embryos, and undoubtedly in many other species of fish [1,32].

Several light pulse experiments, followed by whole transcriptome analysis, have identified that around 20% of all light-induced genes in zebrafish are involved in DNA-repair [51,52]. There is also an enrichment of genes involved in circadian clock entrainment, stress responses, as well as heme metabolism, mitochondrial genes and retinoid binding genes. Furthermore, promoter analysis of these light-induced genes shows an enrichment of E- and D-box enhancers, suggesting these genes use similar signalling pathways as *cry1a* and *per2* [43].

Findings from light-pulsed zebrafish pineal gland transcriptomes were similar to those performed on other tissues, but in addition included transcript targets related to reactive oxygen species (ROS) [50]. Interestingly, the greatest L/D fold difference in light-induced gene expression identified by the pineal transcriptome, is a metabolic gene, 6-phosphofructo-2-kinase/fructose-2,6-biphosphatase 4 (pfkfb4). *pfkfb4* is a target of Hypoxia-inducible factor 1-alpha (hif1a), which is also light induced [50,53]. An implication of this might be that light exposure can directly feed into cellular metabolic regulation.

Most of the results described above relate to directly light-driven transcriptional changes, a fact that extends to the fish brain itself. We know that the fish central nervous system contains many opsins, which are expressed in most, if not all brain regions. The whole cultured fish brain, or regions thereof, can directly respond to light at the transcriptional level. But does this direct brain light detection translate into actual behavioural responses to light? Amazingly, the answer is yes. Eyeless, pineal-less zebrafish larva do indeed change their swimming behaviour in response to light exposure, and the presence of opsins expressed specifically within regions of their brain is responsible for this. Visually blind fish still swim towards light stimuli and perform simple light-seeking behaviour, triggered by loss of illumination [54].

### 3.4. Alternative Fish Models—Cavefish

An alternative to exploring the clock and its downstream biology by “knocking out” key circadian or light-regulated genes, or performing forward genetic mutant screens, is to study “naturally occurring mutants”—especially animals that have adapted to a life in the dark. Two cavefish species, the Somalian blind cavefish (*Phreatichthys andruzzii*) and the Mexican blind cavefish (*Astyanax mexicanus*) have been exploited in this way to study light detection and clock function.

The Somalian cavefish are thought to have been isolated in completely dark underground caves around 2 million years ago [55]. They have typical troglomorphic phenotypes, such as loss of eyes and pigment. The Somalian cavefish have also lost their ability to entrain to artificially provided light-dark cycles, which is possibly due in part to an aberrantly spliced variant of the *per2* transcript which lacks a C-terminal cryptochrome binding domain. This splice variant of PER2 protein is unable to dimerise with CRY and is consequently localised predominantly to the cytoplasm [56]. *P. andruzzii* can, however, entrain to feeding cues, showing a clear anticipatory increase in activity around feeding time. This non-photic zeitgeber also entrains the molecular clock, showing *per1* mRNA rhythms in several organs [4]. Presumably, unlike for light, *per2* is not required for food entrainment of the oscillator. The Somalian blind cavefish expresses two truncated non-visual opsins, melanopsin (*opn4m2*) and a teleost-multiple-tissue opsin (known as *TMT3a*) [4]. These two opsins have been proposed as key photopigments for entrainment. However, as the genome of this cavefish has yet to be sequenced, it is likely that there will be many other candidate opsins to explore. It will be interesting to see how many of the numerous classes of opsins are mutated in this species.

The Mexican blind cavefish, *Astyanax mexicanus*, also have a troglomorphic phenotype. In contrast to *P. andruzzii*, which represents one species and one strain, *A. mexicanus* is the name for 29 unique Mexican cavefish populations, descending from the same ancestral river strain [57,58]. The colonisation of caves is proposed to have happened in two waves in geographically distinct regions, with the oldest isolation event estimated at 2 million years ago [57,59]. The descendants of the founding river species of *A. mexicanus* are still swimming in the local Mexican rivers, making this cavefish a unique and powerful evolutionary and adaptational model. Furthermore, the cave strains and the river strain of *A.mexicanus* have not fully speciated from each other and can still be crossed in the laboratory to produce multiple cave/cave or cave/river hybrid F1 generations.

When studied in the wild, the Mexican blind cavefish show no molecular or behavioural circadian rhythms. However, in contrast to the Somalian cavefish, several of the Mexican blind cavefish strains can entrain to LD cycles under lab conditions, although the phase and timing of *per1* gene expression is altered [2]. Furthermore, light sensitivity is altered in cavefish, with higher basal levels of *cry1a* and *per2a* compared to the surface strains in both adult and embryonic cavefish [1,2]. Interestingly, there are also marked differences between the cave strains, meaning that although they have adapted to similar niches, the specific changes in the remaining clock mechanism is likely to be different. It is possible, therefore, that each cave strain could effectively represent a unique circadian clock and light-responsive mutant, making these animals a very powerful tool for future clock studies.

## 4. The Outputs

### 4.1. Sleep and Rhythmic Behaviour

The topic of sleep regulation is well beyond the scope of this manuscript, and there is a recent, excellent zebrafish sleep review by Oikonomou and Prober [60]. What is already apparent about sleep regulation in zebrafish is that there is a great deal of conservation in the process between teleosts and mammals, with important roles played by the hypocretin system, galanin-containing neurons and pineal melatonin in regulating sleep and activity rhythms [20,61,62,63]. Sleep regulation is always viewed as a two-process model, where a circadian arousal drive is integrated with a homeostatic sleep pressure. With the clock regulating the timing or phase of sleep, and the homeostat regulating the required duration or amount. Of course, this has been classically viewed as there being distinct sleep and circadian centres within the brain and, therefore, much of the effort in sleep research requires the identification of the relevant “sleep circuits”. What has not been taken into account in the context of zebrafish (and possibly also somewhat in mammals) is the fact that circadian pacemakers are now viewed as being highly dispersed within the central nervous system. It is certainly clear in zebrafish that the majority, although possibly not all, neurons have a clock and show widespread direct light-sensitivity [64]. In addition, non-visual photopigments are present throughout most regions of the brain [33]. What this may mean is that the “homeostatic” sleep centres are inherently rhythmic and light responsive themselves, removing the need for any separate “clock circuitry” linking these two brain processes. The sense of time is inherent to the majority of neurons in the brain, and so that aspect of sleep drive is distributed throughout the nervous system. How this may all play out in terms of sleep regulation is for future studies to determine.

### 4.2. The Cell Cycle

One advantage of studying fundamental aspects of cell biology, over perhaps behaviour or physiology, is that the molecular basis of much cell biology is highly conserved across a wide range of species. For example, the basic mechanisms of mitochondrial function, transcriptional regulation and the cell cycle/cell division are very similar whether one is studying human or zebrafish cell cultures. On the whole, the same genes and regulatory proteins appear to be involved, if with some interesting modifications. Studying the cell cycle in an animal like zebrafish should be relatively straightforward due to our molecular understanding from other model systems. Equally, new findings in fish should also be relevant to mammals by the same argument.

It has been well-established for many years that the clock controls the timing of the cell cycle in a wide range of animals and plants, ranging from cyanobacteria to human tissues. In the case of zebrafish cell lines, DNA replication (S-phase) typically occurs in the late evening, and mitosis just before dawn [65,66]. Similar timing is found for the cell cycle in many healthy proliferative human tissues [67,68]. Loosely speaking, there are two conceptual ways in which this might occur. One is that the clock establishes a “gate” or “window” at a specific time of day when cells will pass through a certain stage of the cell cycle. Such a mechanism appears to occur in cyanobacteria and possibly other situations where the cell cycle length is quite short, i.e. is significantly less than 24 h [69]. Another possibility is that the circadian clock entrains the cell cycle by changing its speed, or angular velocity, such that the period of the two oscillators becomes equal. This mechanism appears to occur in at least some mammalian cell lines in culture, where there is a direct coupling of period length between clock and cell cycle [70,71]. This perhaps makes sense in the context of mammalian/human cell division where the cell cycle length is typically close to 24-h.

So, what does zebrafish as a model bring to this particular party? In this case, two aspects of the zebrafish cell system prove to be very useful tools for the study of clock-cell cycle coupling: the direct light entrainment ability of each cell and a very wide “range of entrainment” (Type 0 PRC), allowing for entrainment to a wide range of differing driving light-dark or T-cycles [28,72,73]. Consequently, it is possible to synchronize the cell cycle in zebrafish simply by changing the lighting conditions, a fact that removes the need for pharmacological manipulations to the system [22]. In addition, one can apply a wide range of T-cycles from less than 16 h (8 h light:8 h dark) to 32-h days (16 light:16 dark). The circadian pacemaker can be set to this wide range of periods, and so by imaging cell cycle progression in individual cells, using the FUCCI cell cycle reporter system, it is possible to directly test two hypotheses: (a) the length of the cell cycle changes to match the length of the driving cycle, or (b) the length of the cell cycle remains unaltered, but the timing of a circadian-cell cycle gate changes [73]. Results clearly show that the cell cycle length remains invariant with T-cycle, at a relatively long 45 h. Entrainment of the zebrafish cell cycle, therefore, occurs through a gating process, as has been proposed for cyanobacteria. However, the cell cycle length in zebrafish is much longer. The phase or window of S-phase timing can be clearly shown to alter its phase angle of entrainment to the differing T-cycles. The clock entrains the mechanism that controls the cell cycle gate, allowing cell cycle progression to occur at specific times of day. This gate is not deterministic, but leads to an increased probability that cells will undergo a given cell cycle event at that time, i.e. it is stochastic. In cell culture, cells may “choose” to miss the next window, and a certain percentage will ignore it altogether, but the clock provides a bias in the population, such that cells are more likely to replicate their DNA, and divide at certain times of day.

What is this gating mechanism? A global analysis of well-established cell cycle regulators identified a number of key genes that are under robust circadian clock control. This includes the cell cycle regulator *cyclin-dependent kinase inhibitor 1a* (*CDKN1A* or *p21*), which shows a high amplitude rhythm, peaking around dawn and having a low point of expression in the early evening. *p21* regulates the entry of cells from G1 into S-phase, with high levels of holding cells in G1. The low level of expression at the end of the day corresponds to the time that more cells enter the S-phase from G1. The clock-controlled fall in *p21* levels is the circadian gate or window, which biases the population to proceed with DNA-replication during the dark phase of the day. The promoter, or upstream regulatory region of the *p21* gene contains a number of E-box elements, deletion of which abolishes the circadian rhythm in *p21* expression. Therefore, it does appear that the clock directly controls *p21* as an output gene to set this cell cycle window. Interestingly, correlative data supporting this hypothesis comes from the T-cycle experiments, where it is clear that the phase of the *p21* rhythm changes relative to the entraining light-dark cycle. Importantly, the actual timing of the S-phase also changes under T-cycle conditions, and always matches the low point of *p21* expression—the circadian-cell cycle window for DNA replication [73].

An issue with the evolutionary “design” of the zebrafish clock system is that it is hard to see how one can simply generate internal phase differences between cells and organs, when cells are “all” directly light responsive, and presumably possess the same circadian pacemaker. It is possible that the plethora of photopigments could play some role in this. But how do zebrafish place different processes into different relative times of the daily light-dark cycle? Work on S-phase regulation demonstrates the mechanism that fish probably use, at least in part, to achieve this. A bioinformatic analysis of the zebrafish genome revealed a new, previously unexplored gene that had a high degree of similarity to *p21*, which we subsequently named *p20* [65]. *p20* is also under strong clock control, but the timing of peak expression is shifted by about 6 h relative to *p21*. A promoter analysis reveals that, as for *p21*, *p20* contains critical E-box elements that drive its rhythmic expression. However, inserted amongst these regulatory elements is also an RRE or Rev-erb responsive element. Deletion of this regulatory sequence in the *p20* promoter removes nearly 4 h of the phase difference between the two cell cycle regulators. Interestingly, the spatial expression pattern of these two genes also differs in the zebrafish larvae and adult tissue. *p20* is primarily expressed in the brain, whereas *p21* is primarily expressed in other tissues. An analysis of S-phase timing between brain and intestine reveals a 6-h difference in peak S-phase timing and of course this difference correlates perfectly with the expression patterns of *p20* and *p21*, respectively. Zebrafish generate a difference in the timing of output processes, in this case the S-phase, but employing a) a different but highly related gene and b) by “adding” additional regulatory components to the promoter region of that gene so as to modify phase of expression.

### 4.3. The Clock and Cancer

**Figure 3:**
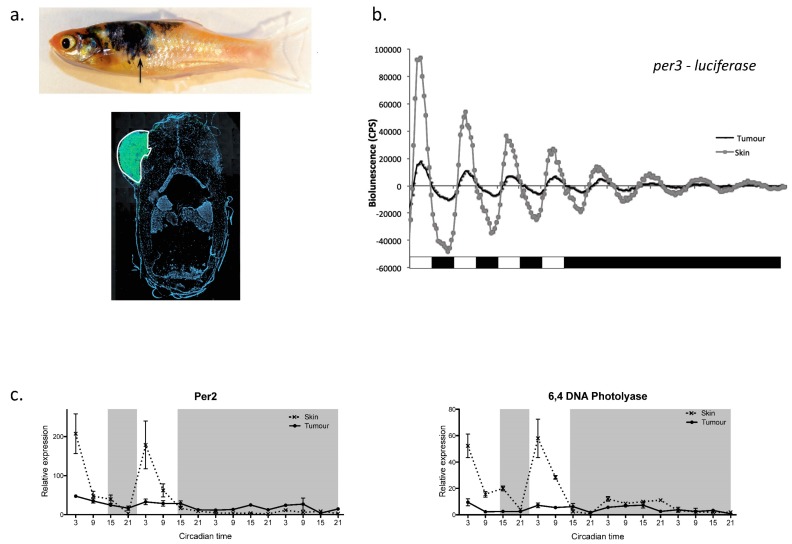
This figure shows specific circadian data collected from a zebrafish melanoma model. (**a**) Transgenic fish were generated, which develop a melanoma in which cells are both GFP-tagged and contain a *per3-luciferase* reporter gene. The lower panel shows a cross-section through a fish revealing the extend of the GFP-positive tumour. (**b**) Luminenescent gene expression recordings from both a developing melanoma and neighbouring skin reveal that circadian gene expression rhythms are significantly lower amplitude in the tumour and damp faster than in healthy skin. (**c**) Two strongly light responsive genes, *Per2* and *6-4 photolyase*, so no transcriptional light induction or rhythmicity in the developing tumour compared to adjacent, healthy skin. (Taken from Hamilton, Diaz-de-Cerio and Whitmore, 2015) [77].

This strong link between the clock and cell cycle regulation raises the obvious question of what role the clock may play in cancer progression. This issue has been explored extensively in mammalian systems, where clock disruption (either through SCN ablation or “jet-lag” protocols) clearly accelerates tumour growth rates [74,75]. There is also an abundance of epidemiological data showing a link between shift work and cancer incidence in humans [76]. Zebrafish have also added to this area of investigation [77]. Using luminescent report animals, containing the *per3-luciferase* gene, it is possible to monitor clock function in a developing melanoma, versus that in neighbouring healthy tissue. Figure 3 summarises some of the data collected using this approach. In a zebrafish melanoma model, it is absolutely clear that the amplitude or robustness of the *per3* rhythm is dramatically reduced—the circadian pacemaker is simply not working as well in the tumour. A consequence of this is that the daily rhythm in mitosis within the melanoma is lost, such that cells now divide randomly across the day-night cycle. This corresponds to a loss in the circadian regulation of the underlying *cyclin B1* transcriptional oscillation, which underpins this mitotic rhythm. Interestingly, in the context of zebrafish melanoma, the first detectable changes in clock regulation all relate to the direct light responsiveness of the tumour. The acute light induction of all light-responsive genes appears to be lost or at least greatly reduced. Genes, like *cry1a* and *per2*, that are key to fish clock entrainment are barely light activated, which may well explain why the amplitude of oscillations in the melanoma are so low. Interestingly, the light activation of DNA repair genes is also inhibited within the melanoma. This fact may also be a key aspect as to why circadian disruption leads to accelerated tumour growth. Not only do cells in the tumour lose their daily, time restricted rhythms in mitosis, dividing at all times of the day, but they also lose the ability to repair additional (possibly light) induced mutations. Cells undergo active mitosis during the day, and then can not repair the additional DNA damage that might occur due to UV exposure, accelerating tumour progression even further. It will be very interesting to see how this scenario plays out in human melanoma development, when such studies are performed.

## 5. Conclusions

In this short review we hope we have discussed the more novel contributions that zebrafish and certain other teleosts have made to our understanding of circadian biology, particularly focusing on unique aspects of clock entrainment as well as downstream, output regulation. This is, of course, only a small part of the teleost circadian field and has missed out excellent studies from a wide range of other species of fish and research groups. The strength of teleosts lies very much in the wide variety of species, over 30,000, of fish that exist. They have solved the evolutionary problem of how to live on a rhythmic planet in a mass of varied ways, and as such offer a remarkable resource for studying environmental adaptation at a molecular/genetic level. Moreover, many fish species are of considerable commercial significance, and the importance of fisheries/fish farming and fish stocks is of major international interest. Laboratory mice have not quite yet found their way to our supermarket shelves, and so studies of clock biology in “large” fish is not only of interest, but of significant economic value. There is some excellent work in this area that this review does not have space to discuss, but clearly this is a topic of major future importance. It is also worthy of note that most fish species are diurnal, although in fact many can switch between nocturnal and diurnal as environmental situations and seasons change. As a diurnal vertebrate model, as opposed to a nocturnal rodent, there may be much to learn from zebrafish clocks and their entrainment that relates to human clock function. The core clock mechanism of fish is still relatively unexplored, but there may yet be interesting surprises to uncover in that area as “time” progresses.
